# Transcultural differences in suicide attempts among children and adolescents with and without migration background, a multicentre study: in Vienna, Berlin, Istanbul

**DOI:** 10.1007/s00787-021-01805-7

**Published:** 2021-05-29

**Authors:** Zeliha Özlü-Erkilic, Robert Diehm, Thomas Wenzel, R. Hülya Bingöl Ҫağlayan, Hatice Güneş, Özden Şükran Üneri, Sibylle Winter, Türkan Akkaya-Kalayci

**Affiliations:** 1 Department of Child and Adolescent Psychiatry, Outpatient Clinic of Transcultural Psychiatry and Migration Induced Disorders in Childhood and Adolescence, Medical University of Vienna, Währinger Gürtel 18-20, 1090 Vienna, Austria; 2 Department of Pediatrics and Adolescent Medicine, Medical University of Vienna, Währinger Gürtel 18-20, 1090 Vienna, Austria; 3 Department of Psychiatry and Psychotherapy, Medical University of Vienna, Währinger Gürtel 18-20, 1090 Vienna, Austria; 4 Department of Child and Adolescents Psychiatry, Cerrahpaşa School of Medicine, İstanbul University-Cerrahpaşa, Kocamustafapasa Cd. No: 53, Fatih, Istanbul, Turkey; 5 Department of Psychology, İstanbul Gelisim University, Cihangir Mahallesi Şehit Jandarma Komando Er Hakan Oner Sk. No:1, Avcilar, Istanbul, Turkey; 6 Department of Child and Adolescent Psychiatry, Bakirkoy Training and Research Hospital for Mental Health and Neurological Disorders, Zuhuratbaba Mah. Dr Tevfik Sağlam Cad. No:25/2, Bakirköy, Istanbul, Turkey; 7 Department of Child and Adolescents Psychiatry, Ankara City Hospital, AYBÜ Ankara Şehir Hastanesi Çocuk Hastanesi 06800 Bilkent, Ankara, Turkey; 8 Departement of Child and Adolescent Psychiatry, Psychsomatics and Psychotherapy, Charité-Universitätsmedizin Berlin, Campus Virchow, Augustenbruger Platz 1, 13353 Berlin, Germany; 9 Postgraduate University Program Transcultural Medicine and Diversity Care, Medical University of Vienna, Spitalgasse 23, 1090 Vienna, Austria

**Keywords:** Transcultural differences, Suicide attempts, Children and adolescents, Risk factors, Migration background

## Abstract

While suicide can occur throughout the lifespan, worldwide suicide is the second leading cause of death among young people aged between 15 and 29 years. The aim of this multicentre study, conducted in Austria, Germany and Turkey, is to investigate the transcultural differences of suicide attempts among children and adolescents with and without migration background. The present study is a retrospective analyses of the records of 247 young people, who were admitted after a suicide attempt to Emergency Outpatient Clinics of Departments of Child and Adolescent Psychiatry of the collaborating Universities including Medical University of Vienna, Charité University Medicine Berlin and Cerrahpaşa School of Medicine and Bakirkoy Training and Research Hospital for Mental Health in Istanbul over a 3-year period. The results of the present study show significant transcultural differences between minors with and without migration background in regard to triggering reasons, method of suicide attempts and psychiatric diagnosis. The trigger event “intra-familial conflicts” and the use of “low-risk methods” for their suicide attempt were more frequent among patients with migration background. Moreover among native parents living in Vienna and Berlin divorce of parents were more frequent compared to parents living in Istanbul and migrants in Vienna. These results can be partly explained by cultural differences between migrants and host society. Also disadvantages in socio-economic situations of migrants and their poorer access to the healthcare system can mostly lead to acute and delayed treatments. Larger longitudinal studies are needed to understand better the impact of migration on the suicidal behaviour of young people.

## Introduction

Around the world, about 800,000 people die by suicide yearly [1]. Suicide is a phenomenon, which occurs throughout the lifespan, but worldwide suicide is the second leading cause of death among young people aged 15–29 years [2]. The risk of death due to suicide enhances with increasing age, but suicide attempts are more common among adolescents [3]. Also suicidal ideation and suicide attempts are quite common among younger people around the globe [4–8].

In the European Union, more than 55,000 suicides occurred each year [9]. While in 2016 the age-standardized suicide rate was worldwide given as 10.53 (suicides per 100,000 population), the age-standardized suicide rates according to WHO were 11.4 (male: 17.5; female: 5.7) in Austria, 9.1 (male: 13.6; female: 4.8) in Germany and 7.2 (male: 11.3; female: 3.2) in Turkey [10]. In 2018, death due to suicide was the second leading cause among 15–20 year olds living in Austria [11]. In Germany, a lifetime history of suicide attempts in adolescents was reported between 6.5 and 9.0% by previous studies [12–14]. Therefore suicidality is a major public health concern especially among adolescents [15]. As a consequence, in European countries, policy focus on and public interest in suicidal behaviour is considerably high, so that suicide research is recently getting more and more attention [16]. There are different risk factors, which increase suicidal behaviour among children and adolescents [17, 18]. For example, migrants as a vulnerable population group have a higher risk for suicidal behaviour [19]. Researches in European countries showed that migration status increases the risk of suicide attempts among adults [20–22] as well as among adolescents [23, 24] as migration-induced stress is in general a risk factor for mental health problems [25–27]. As a consequence specific migration-related stress factors such as acculturation stress, racist and xenophobic politics and socio-economic disadvantages increase the vulnerability of the migrant population for mental health disorders in general but also the risk of suicidal behaviour [28–32]. These migration-related stress factors affect particularly vulnerable children and adolescents [23, 33–40]. Accordingly, the study of Donath et al. 2019 reported that migrant adolescents had about 1.5 times higher risk for suicidal behaviour than their native counterparts [41]. In addition, the physical [42] and mental health [34, 43–47] of migrant children and adolescents is more frequently impaired than in their native peers.

The study of Grube 2004 reported that three main factors: migration status, young age and female gender increase the risk of suicide [20]. Similarly, the study of Yilmaz et al. 2008 showed that suicide rates among Turkish-speaking female young migrants are higher than that of their male peers [22] as well as compared to that of their native peers [48]. Also the suicide rate among Turkish-speaking adult migrants living in some European countries is higher than that of the native population [9]. Furthermore, suicidal behaviour is more frequent among children and adolescents with low socioeconomic status [49]. Migrants living in Germany and Austria, in general, have a much lower income level and suffer more frequently from poverty and mental disorders as compared to the native population [50, 51]. In addition, they experience higher barriers in access to the healthcare system in Germany and in Austria as compared to the native population [51, 52].

The study of Akkaya-Kalayci et al. 2017 showed that the rate of suicide attempts among Turkish-speaking children and adolescents, who had an acute treatment at the psychiatric emergency clinic, was noticeable higher as compared to their native Austrian and Serbian/Bosnian/Croatian peers [50].

### Aim of the study

The aim of the present multicentre study is to analyse the transcultural differences of suicide attempts between native children and adolescents and their peers with migration background. We conducted a multicentre study in the metropolitan capitals of three countries, Austria, Germany and Turkey. A further aim of the present study was to analyse differences in suicide attempts in the subgroups of Turkish-speaking minors with migration background living in Berlin and Vienna and those of their peers without migration background living in Istanbul.

## Methods

This study was accomplished in Austria, Germany and Turkey between June 2011 and June 2014. We conducted our study in metropolitan areas of three different countries, i.e. Austria, Germany and Turkey, which share a similar socio-demographic structure. The proportion of migrants living in Vienna (45.2%), Berlin (33.4%) and Istanbul (35.7%) are correspondingly quite high. Indeed in Berlin and Istanbul almost every third and in Vienna nearly every second person has a migration background. Furthermore, the proportion of Turkish-speaking migrants living in Berlin and Vienna is quite high. In Berlin, Turkish-speaking people are the largest and in Vienna the second largest migrant group [11, 53], Turkish Statistical Institute, 2018). Therefore, we included Turkish-speaking migrants living in Berlin and Vienna for our comparative study and focused on transcultural differences between young native and Turkish-speaking suicide attempters.

We retrospectively analysed the patient records of in total 247 children and adolescents, who were admitted after a suicide attempt to an Emergency Outpatient Clinic of the respective University hospitals, located in Vienna, Berlin and Istanbul over a 3-year period (Hospital of the Medical University of Vienna, Charité University Hospital Berlin and Cerrahpaşa School of Medicine, University of Istanbul and Bakirkoy Training and Research Hospital for Mental Health and Neurological Disorders, Istanbul). We systematically collected relevant data such as socio-demographic features (e.g. gender, age, nationality, living city, family situation, generation of migration, etc.) and psychiatric diagnoses of the suicide attempters from their patients file, based on a matrix of factors identified as relevant in earlier research (see introduction). In addition, we collected detailed information about the suicide attempts (e.g. reported trigger of the suicide attempt, method of the suicide attempt, prior verbal communication of suicidal ideation), if available from the records. We further compared a specific important subgroup (Turkish-speaking patients living in Berlin, Vienna and Istanbul) to explore if migration background and the duration of the migration have a relevant and important influence on their suicide attempts.

Previous studies demonstrated that the use of emergency interventions among migrants is higher [54, 55] compared to the native population, which may be due to cultural and institutional barriers to early intervention and preventive measures that might lead to delayed care [55]. Therefore, we analysed retrospectively the patient files of children and adolescents, who were treated at the emergency departments after attempting suicide.

Migration background is defined as “at least one parent (second generation) or grandparent (third generation) was not being born in the host country” [56, 57].

In the present study, the ethnicity of the patients from Turkey was not recorded in detail. Therefore in the context of this article the term “Turkish-speaking minors” is used to define all the minors originating from Turkey and it does not refer to mother language, main language used in everyday life or to primary citizenship, as some of them may have other mother tongue than Turkish as well different ethnic backgrounds (e.g. Kurds, Arabs, etc.) and official citizenship.

The definition of suicide attempt is “engagement in potentially self-injurious behaviour with at least some intent to die” [58].

Statistics evaluation was performed using IBM SPSS Statistics, version 24. Differences between groups in nominal data were calculated by Chi-Square tests considered with an α-error of 5%. For odds ratios, binary logistic regression analyses were calculated with the group of natives in Istanbul being baseline and dummy variables for each of the other four groups.

Prior approval was obtained from the Ethics Committees of the participating Universities and are on record with the authors.

## Results

### Study sample

In the present study, we analysed the patient records of in total 247 children and adolescents aged between 10 and 18 years. The study subjects were living in Vienna, Berlin or Istanbul and had received outpatient emergency treatment after attempting suicide in the departments of child and adolescents psychiatry in one of the participating hospitals. All consecutive such admissions were included in the study.

In line with previous studies [59–62], the majority of the suicide attempters were female in all study groups. The study population in Berlin even consisted of only female patients. Therefore, the gender differences within each study groups were highly significant (*χ*^2^ = 89.134; *df* = 4; *p* < 0.001). In all groups, significantly more females than males attempted suicide.

The mean age of the study groups was quite similar, i.e. between 15.31 and 15.93 (SD = 1.9).

Between the study groups in the three centres no significant age [*χ*^2^(4) = 18.528; *p* = 0.422] or gender [*χ*^2^(4) = 8.794; *p* = 0.066] differences were found.

Therefore the groups of the present study are similar in age and gender with no artefacts expected in these variables (Table 1).

**Table 1:** Study sample: children and adolescents, admitted after a suicide attempt in the participating hospitals

	Males		Females		Mean age		Total	
	*N*	(%)	*N*	(%)	MD	SD	*N*	(%)
Native minors in Berlin	3	12.5	21	87.5	15.6	1.4	24	100
Turkish-speaking minors in Berlin	0	0	22	100	15.8	1.6	22	100
Native minors in Vienna	23	25.8	66	74.2	15.3	2.2	89	100
Turkish-speaking minors in Vienna	8	26.7	22	73.3	15.9	1.2	30	100
Native minors in Istanbul	19	23.2	63	76.8	15.9	2.7	82	100
Total	53	21.5	194	78.5	15.7	1.9	247	100

### Trigger of the suicide attempts

In all study groups “interfamilial conflicts” were the leading reported trigger of suicide attempts (38.9%, see Tab. 2). However, there were differences in regard to the frequency of triggering events between children and adolescents with and without migration background. Among patients with migration background suicide attempts reportedly caused by “intra-familial conflicts” differed significantly between the four groups [*χ*^2^(4) = 21.81, *p* < 0.01]. While about one third of the native minors living in Berlin (37.5%), Vienna (38.2%) and Istanbul (30.5%) attempted suicide due to “intra-familial conflicts”, more than half of the migrant minors living in Vienna (53.3%) and Berlin (54.5%) had “intra-familial conflicts” as reported trigger events (Table 2).

**Table 2:** Reported trigger of the suicide attempts

	Native minors in Berlin		Turkish-speaking minors in Berlin		Native minors in Vienna		Turkish-speaking minors in Vienna		Native minors in Istanbul		Total		*χ*^2^	*p*
	*N*	(%)	*N*	(%)	*N*	(%)	*N*	(%)	*N*	(%)	*N*	(%)		
Intra-familial conflicts	9	37.5	12	54.5	34	38.2	16	53.3	25	30.5	96	38.9	21.81	< 0.01
Relationship/separation crisis	6	25.0	3	13.6	15	16.9	6	20.0	20	24.4	50	20.2	20.60	< 0.01
Critical life events	2	8.3	4	18.2	3	3.4	1	3.3	5	6.1	15	6.1	*	
Problems in school/with the peer group	2	8.3	2	9.1	19	21.3	4	13.3	12	14.6	39	15.8	*	
Mental illness	3	12.5	0	0	13	14.6	0	0	13	15.9	29	11.7	*	
Unknown reason	2	8.3	1	4.5	5	5.6	3	10.0	7	8.5	18	7.3	*	
Total	24	100	22	100	89	100	30	100	82	100	247	100		

*Not possible because of low cell count

The second most common trigger for a suicide attempt was again quite different between the study groups [*χ*^2^(4) = 20.60, *p* < 0.01]. Relationship/separation crisis of the patients from their partner were the second leading cause for suicide attempts among natives living in Berlin (25.0%) and Istanbul (24.4%) and in Turkish-speaking minors living in Vienna (20%). Among Turkish-speaking minors living in Berlin (18.2%) “critical life events” and among natives living in Vienna (21.3%) “problems in school/with the peer group” were the second most commonly reported trigger for a suicide attempt.

### Methods of the suicide attempts

In the present study, we clustered the methods of suicide attempts reported from the study sample in low-risk and high-risk categories according to the ICD-10 Classification. Low-risk methods are drug overdose (X60–X64) and cuts/stabs (X78). Whereas jumping from heights (X80), shooting (X72–X74), being run over (X81) and drowning (X71) were defined as high-risk methods due to their high risk of lethal outcome [63].

When analysing by migration background and lethality of means, records were found to be quite complete, as the information about the chosen methods of the suicide attempt was missing among only 6 patients. 4 patients chose both, low-risk as well high-risk methods for their suicide attempt. With exception of the Turkish-speaking minors living in Berlin, one person in each group chose both methods for his/her suicide attempt.

Between the study groups, significant differences were present by the chosen method of suicide (*χ*^2^ = 19.622; *df* = 8; *p* < 0.012). Most study groups choose the low-risk methods *“drug intoxication”* for their suicide attempts. However, Turkish-speaking patients living in Vienna (71.4%), Berlin (76.2%) and Istanbul (79.7%) had the highest rate of drug intoxication compared to other study groups; generally the percentages were about three quarters. On the other side, only about half of the natives living in Vienna (54%) and 63% of the natives living in Berlin attempted suicide by the more lethal “drug overdose”. The high-risk method “jumping from height” is the second common method for suicide attempts in the whole study sample (11.8%). In general, children and adolescent with migration background chose more low-risk methods while native patients (patients without migration background) used more high-risk methods for their suicide attempt (Table 3). In general, high-risk methods where used in a much higher percentage of native minors in both Vienna (42.5%) and Berlin (33.3%).

**Table 3:** Methods of the suicide attempts among study groups (*p* < 0.012)

	Low-risk methods		High-risk methods		Total	
	*N*	(%)	*N*	(%)	*N*	(%)
Native minors in Berlin	15	62.5	8	33.3	24	100
Turkish-speaking minors in Berlin	20	90.9	2	9.1	22	100
Native minors in Vienna	49	56.3	37	42.5	87	100
Turkish-speaking minors in Vienna	21	75.0	6	21.4	28	100
Native minors in Istanbul	64	80	15	18.8	80	100
Total	169	70.1	68	28.2	241	100

### Psychiatric diagnosis groups according to ICD-10 classification of the suicide attempters

Information about the respective psychiatric diagnosis group recorded for admission was available in the medical records of 186 patients.

In the whole study sample, the psychiatric diagnostic categories of mood (F3) (36.6%) and F 4 (stress related, somatoform and neurotic) disorders (27.4%) were the most frequent.

In all study groups, except among the Turkish-speaking minors living in Vienna the F3 (mood (affective) disorders) were the most common diagnostic categories. However, among the Turkish-speaking minors living in Vienna F4 (neurotic, stress-related and somatoform disorders) was the most common psychiatric diagnosis group, reported in 41% of the patients.

There were significant differences between the study groups in psychiatric diagnosis categories F3 [*χ*^2^(4) = 43.18; *p* < 0.01], F4 [*χ*^2^(4) = 19.88, *p* < 0.01] and F9 [*χ*^2^(4) = 21.29, *p* < 0.01] (see Table 4).

**Table 4:** Psychiatric diagnosis according to ICD-10 classification of the patients per group

	Native minors in Berlin		Turkish-speaking minors in Berlin		Native minors in Vienna		Turkish-speaking minors in Vienna		Native minors in Istanbul		Total		*χ*^2^	*p*
	*N*	(%)	*N*	(%)	*N*	(%)	*N*	(%)	*N*	(%)	*N*	(%)		
F1	2	11.8	3	23.1	3	4.2	2	9.1	1	1.6	11	5.9		*
F2	0	0	0	0	1	1.4	0	0	1	1.6	2	1.1		*
F3	7	41.2	5	38.5	18	25.4	5	22.7	33	52.4	68	36.6	43.18	< 0.01
F4	3	17.6	4	30.8	16	22.5	9	40.9	19	30.2	51	27.4	19.88	< 0.01
F5	1	5.9	0	0	1	1.4	0	0	0	0	2	1.1		*
F6	1	5.9	0	0	16	22.5	0	0	5	7.9	22	11.8		*
F7	0	0	0	0	0	0	1	4.5	0	0	1	0.5		*
F8	0	0	0	0	1	1.4	0	0	0	0	1	0.5		*
F9	3	17.6	1	7.7	15	21.1	5	22.7	4	6.3	28	15.1	21.29	< 0.01

*χ^2^-test not possible because of low cell count

While more than half (52.4%) of the native minors living in Istanbul had received a F3 diagnosis, Berlin natives (41.2%) as well Turkish-speaking minors (38.5%) in the same city had a similarly frequent diagnosis of F3. Only about a quarter of Viennese native (25.4%) as well Turkish-speaking minors (22.7%) in Vienna had received the psychiatric diagnosis of F3. Nearly 41% of the Turkish-speaking minors living in Vienna but only about a third of their ethnic peers in Berlin (30.8%) and in Turkey (30.2%) were diagnosed by F4. On the other side, natives living in Berlin (17.6%) and Vienna (22.5%) had less frequently received a F4 diagnosis compared to their Turkish-speaking peers in three centres. Although F9 diagnosis was most frequently recorded similarly among native (21.1%) and Turkish-speaking minors (22.7%) living in Vienna, a F9 diagnosis was comparatively rare among Turkish-speaking minors living in Istanbul (6.3%) and in Berlin. (7.7%). (Table 4).

### Family situation

**Fig. 1:**
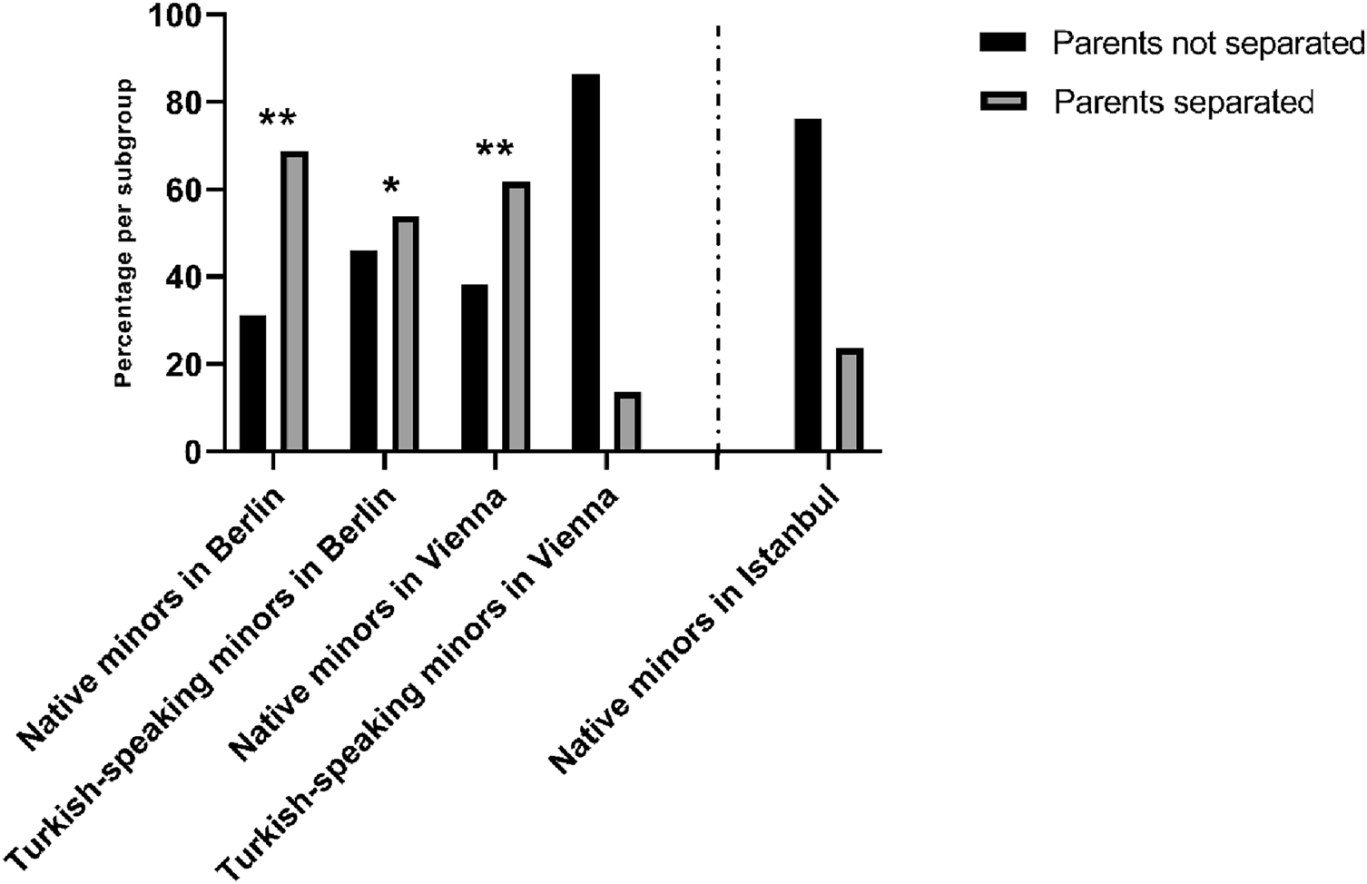
Relationship status of patient’s parents per subgroup. Percentage per subgroup of patients with parents who are separated or not. *p < 0.05, **p < 0.01, indicating significant OR in logistic regression analyses with native minors in Istanbul being the baseline

Records on the relationship status of their parents were available for 165 patients (66.8%). Most of the parents of native patients living in Berlin (68.8%, *n* = 11) and in Vienna (61.8%, *n* = 34) were separated. In contrast, only about one quarter of the parents of native minors living in Istanbul (23.7%, *n* = 14) were separated. The rate of separated parents in the immigrated groups of Turkish-speaking minors in Berlin was in comparison 53.8%, *n* = 7. The separation rate of Turkish-speaking parents living in Vienna (13.6%, *n* = 3) was even lower than that of their native counterparts in Istanbul (Fig. 1).

The calculated logistic regression model with native minors in Istanbul used as baseline showed a good likelihood ratio test [*χ*^2^(4) = 31.16; *p* < 0.01] and good fitness test with Hosmer & Lemeshow [*χ*^2^(3) < 0.01; *p* > 0.99]. Cox and Snells *R*^2^ was 0.172 with Nagelkerke’s *R*^2^ = 0.232.

The odds ratio of their parents being separated were OR = 7.01 [95% CI 2.10–23.84; Wald’s *χ*^2^(1) = 9.95; *p* < 0.01] for natives in Berlin. The odds ratio for Turkish-speaking families in Berlin was OR = 3.75 [95% CI 1.08–13.02; Wald’s *χ*^2^(1) = 4.33; *p* = 0.04]. For natives in Vienna, the odds ratio was OR = 5.20 [95% CI 2.32–11.70; Wald’s *χ*^2^(1) = 15.94; *p* < 0.01] and for Turkish-speaking families in Vienna the odds ratio was OR = 0.51 [95% CI 0.13–1.97; Wald’s *χ*^2^(1) = 0.96; *p* = 0.33].

### Disclosure of suicidal ideation

In total data of 148 children and adolescents (59.9%) on whether they communicated their suicidal ideation or kept it secret before suicide attempt were available for analyses.

In the whole study sample, the majority (65.5%, *n* = 97) of patients had communicated their suicidal ideation before they attempted suicide.

Natives living in Berlin (66.7%, *n* = 12) and Vienna (93.3%, *n* = 56) most commonly communicated their suicidal ideation, whereas only 34.6% (*n* = 18) of the natives living in Turkey talked to others about their suicidal tendencies.

**Fig. 2:**
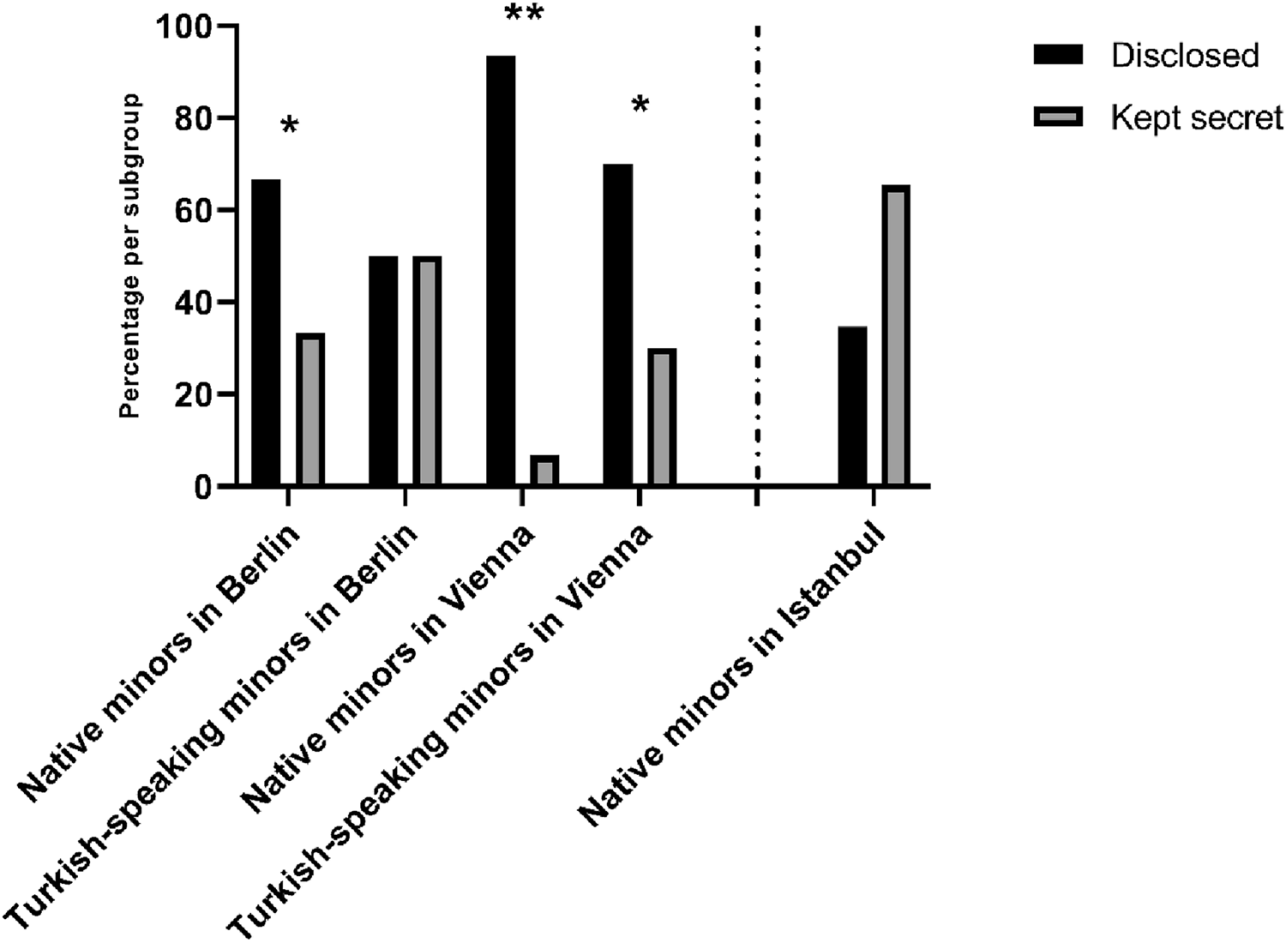
Disclosure of suicidal ideation per subgroup. Percentage per subgroup of patients who did communicate their suicidal ideation before the suicide attempt. *p < 0.05, **p < 0.01, indicating significant OR in logistic regression analyses with native minors in Istanbul being the baseline

Only half of the Turkish-speaking patients living in Berlin (50.0%, *n* = 4) communicated their intent prior to the suicide attempt, whereas 70.0% (*n* = 7) of the Turkish-speaking underaged migrants living in Vienna talked to others about their suicidal ideation (Fig. 2).

The likelihood ratio test for the logistic regression model showed a good value for comparison with the basic model [*χ*^2^(4) = 47.94; *p* < 0.01]. The model test of Hosmer & Lemeshow [*χ*^2^(3) < 0.01; *p* > 0.99] showed a good fit for the model. Cox and Snells *R*^2^ was 0.277 with Nagelkerke’s *R*^2^ = 0.382.

Odds ratio for disclosure was OR = 3.78 [95% CI 1.22–11.75; Wald’s *χ*^2^(1) = 5.27; *p* = 0.02] for natives and OR = 1.89 [95% CI 0.42–8.46; Wald’s *χ*^2^(1) = 0.69; *p* = 0.41] for Turkish-speaking minors living in Berlin. In Vienna, the odds ratios were OR = 26.44 [95% CI 8.26–84.71; Wald’s *χ*^2^(1) = 30.40; *p* < 0.01] for native minors and OR = 4.41 [95% CI: 1.02–19.14; Wald’s *χ*^2^(1) = 3.92; *p* = 0.05] for youth who had Turkish origin.

## Discussion

The results of the present study show noticeable, but complex transcultural differences in the background and form of suicide attempts between migrant and non-migrant children and adolescents living in Austria, Germany and Turkey. Additionally, obvious differences were observed among Turkish-speaking minors with migration background living in different host countries (Austria vs. Germany), as the migration history especially the duration of stay and the influences in the host country are quite different.

In line with previous studies also in the present study [59–61], the number of female exceed those of male suicide attempters in all groups. While, suicide attempts are more common among females [58, 65–69] more males [70, 71] die by virtue of completed suicide. According to the World Health Organisation (1999), globally male individuals commit 2–3 times more suicides than females, whereas suicide attempts are more frequent among women compared to men. Males usually choose high-risk methods for their suicide attempts, which most frequently end lethal [72].

In the present study, evident transcultural differences in regard to the triggering events for the suicide attempts were present. While more than half of the minors with migration background attempted suicide due to “intra-familial conflicts”, just about one-third of the native minors living in Berlin, Vienna and Istanbul attempted suicide reportedly after conflicts within the family. There was also a difference between Turkish-speaking minors living in Vienna, Berlin and the minors in Istanbul, as Turkish-speaking minors with migration background attempted suicide more frequently due to “intra-familial conflicts” than the minors without migration background in Istanbul indicating that this factor should receive more attention especially, though not only, in migrant families. These results are in line with previous studies, [65, 73], which report more “intra-familial conflicts” as common problems within migrant families as compared to those without migration backgrounds. Indeed conflicts within the family can be an important risk factor for suicidality among adolescents [74, 75]. Moreover, studies show that particularly dysfunctional familiar communication can lead to increased suicidal behaviour among adolescents [76–79].

The results of the present study can be partly explained with cross-cultural differences among Austrians, Germans and Turkish-speaking minors originating from Turkey. Similar as many European countries, Austria and Germany, are individualistic (particularistic) and egalitarian societies, while in contrast Turkish culture similar to other Asian societies values group cohesion, collectivism and patriarchal attitudes [23, 50, 65, 73, 80] and can be described also as “universalistic” [81]. Individualism and collectivism (or in different models, particularistic and universalistic cultures) are quite different in many aspects of lifestyles, values and family functioning [82]. Furthermore, in European countries migrant families originating from Turkey are predominantly hierarchically and traditionally oriented. The Turkish populations, in general, can be seen as a collectively oriented culture and the majority of the Turkish population are (Sunni) Muslims. Therefore, Turkish-speaking minors living in European countries probably suffer more from acculturation stress as substantial cultural and religious differences must be faced between the culture of origin and the dominant host [23, 50, 65]. We assume that because of these cultural differences intergenerational conflicts during puberty are more intensive in at least Turkish migrant families [83] leading obviously to more intra-familial disputes. These cultural differences between the values of migrant families and attitudes of the host society can be expected to lead to desperate exit strategies in children and adolescents that have difficulties at the same time adapt to various aspects of different and often conflicting lifestyle aspects [23].

Our results show that native minors more frequently chose high-risk methods; in contrary migrant minors used significantly more low-risk methods for their suicide attempts, which can be seen as characteristic for this group and could be seen as cultural idiom of distress in this groups. Among Turkish-speaking patients living in Vienna, Berlin and Istanbul medical drug intoxication for attempting suicide, which is a low-risk method, was the most frequent method, used in about 71–80% of this group. We assume that suicide attempts using medical drugs are more frequent among youths with migration background compared to their indigenous peers. This can be explained with the drug use or abuse patterns, especially of analgesics, by migrant parents [84] that might, in turn, lead to an easier access of diverse drugs for children and adolescents with migration background at home. This fact can also explain the differences between the chosen method of suicide attempters with and without migration background in our data. The study of von Ferber et al. [85] showed that utilisation of medical drugs is more common among Turkish-speaking migrants compared to the native German population. Especially analgesics were more commonly used by Turkish-speaking migrants as compared to the native population probably due to the high frequency of psychosomatic disorders among this ethnic group [85].

In the present study, a diagnosis from the F3—mood (affective) disorders ICD 10 group was the most common psychiatric diagnosis among native minors living in Berlin, Vienna and Istanbul and Turkish-speaking minors in Berlin. Only among Turkish-speaking minors living in Vienna F4 (neurotic, stress-related and somatoform disorders) was most frequently diagnosed. It should be considered, that in suicidal behaviour a larger range of psychological, social, biological, cultural and environmental factors can play crucial roles. While psychiatric disorders are the strongest predisposing factors for suicidal behaviour [86, 87] in Europe and in North America, on the other hand impulsiveness and not any specific Psychiatric diagnosis is seen as the decisive aspect of suicidal behaviour in Asian societies [88, 89]. The records of native patients in Berlin as well in Vienna and in Istanbul and Turkish-speaking migrants in Berlin included more diagnoses of affective disorders as compared to that of Turkish-speaking migrants living in Vienna. We suppose that the adaptation of the Turkish-speaking migrants in Berlin to the attitudes of the German host culture is stronger than that of their compatriots living in Austria to local culture, as the Turkish-speaking migrant community living in Germany has a longer migration history. In the early 1960s, the migration flow of Turkish-speaking “guest workers” began to Germany (1961) as well to Austria (1964). Nevertheless, Austria was not an attractive host country for “guest workers” due to low salaries; therefore, at the beginning noticeably fewer Turkish-speaking people migrated rather to Germany then to Austria [90, 91]. Therefore, at present, young migrants living in Austria predominantly belong to the second generation and their peers living in Germany rather than the third generation of migrants [53, 92]. The longer the duration of stay abroad the more adaptation to the attitudes of the host country can be expected, so that the preferences and behaviours, including health-related behaviours of the migrants adapts to that of the host country [93]. Due to different migration history, in the present study more Turkish-speaking patients living in Berlin (35.7%) were belonging to the third generation migrants as compared to their compatriots living in Austria (8%).

In the present study population, the rate of “broken homes” among natives living in Vienna and Berlin was higher compared to Istanbul. Additionally, Turkish-speaking parents living in Berlin had a higher rate of divorce/separation compared to Turkish-speaking parents living in Vienna. In line with our results, also the study of Eskin et al. reported increased suicidal ideation among individuals from divorced parents [94]. In the study of Eskin et al. [94] significantly more Austrian parents (26%), were separated compared to Turkish-speaking parents (5.8%). Rates in the Austrian families were therefore nearly four times that of the local Turkish families living in Austria. Both Austria and Germany are as noted before individualistic oriented (particularistic) countries, where divorce/separation is more common. In contrast, Turkey is a mainly universalist culture, characterized by collectivism, in which family cohesion has high importance, therefore, getting divorced/separated is still not so common in the Turkish community [95]: in 2017, the rate of divorce in Turkey (21%) was noticeable lower compared to Austria (41%) and Germany (38%) [11, 53, 96]. We assume that, in the present study populations, the Turkish-speaking parents living in Berlin might have adapted to a higher degree to the attitudes of the host country due to their longer stay time in Berlin. Therefore their rate of divorce is higher compared to Turkish-speaking parents living Vienna.

In our study, natives living in Vienna and Berlin most commonly communicated their suicidal ideation, whereas natives in Istanbul most frequently did not talk about their suicidal tendencies. Turkish-speaking minors living abroad most commonly expressed their suicidal ideation; in contrary, their compatriots living in Turkey kept their suicidal tendencies mostly secret. In collectively oriented countries, talking about emotions or psychological problems is culturally uncommon; due to fear of “loss of face” therefore they mostly emphasize physical complaints (cultural formulation). [85, 97]. As people from collectivistic cultures rather do not tolerate diversity and deviation from the norms, they are more likely to hold stigmatising attitudes toward mental problems compared to people from individualistic cultures [98–100]. Additionally, children and adolescents usually adapt faster to the host country environment [101]. These facts may, as noted, explain the differences in our study in openness and communication of suicidal ideation between the Turkish-speaking youths living abroad and in the respective countries of origin. This might be a crucial factor to be considered in prevention and early intervention strategies in regard to suicide.

It has been reported that migrants living in European countries like Germany have in general higher rates of suicidal behaviour, which may be due to acculturation stress caused by adapting oneself to a highly different culture, help-seeking and also due to higher barriers to the use of health care services [52]. In the study of Grube, 2004 a “transcultural conflict” has been reported by about 71% of the migrants who committed suicide [20]. Especially migrant youths are in general a group vulnerable to mental health problems and suicidal behaviour because of such transcultural conflicts and in consequence must be expected to have special treatment needs. Therefore diversity-care measures are of the utmost importance. Unfortunately in many European countries diversity-care offers which meet culture and language-sensitive needs of migrant youths are not well established in the health care system. Therefore health care systems in countries with a high migrant population need experts with transcultural competences to be able to adequately treat suicidal youths with migration background [38, 102, 103]. Further better communication by native speaker health care professionals and if not available, professional health care translators and video translation must be made available as good communication and trust can be seen as crucial in early recognition of suicidal ideation.

Studies in different European countries: Sweden, London, Belgium and Germany reported that migrants usually had poorer access to preventive health care offers compared to the respective native populations [104, 105]. Consequently due to infrequent use of prevention programmes they are overrepresented in emergency units [50, 54]. Unfortunately at the same time many European countries still are not able to grant full equality in health care access for migrants [38] although the proportion of migrants living in Europe is quite high. However, previous studies show that interventions with greater family support such as skills training of parents to better deal with self-harming behaviour of their suicidal children could effectively reduce the self-harming behaviour of adolescents [87, 106]. Moreover, functional communication between adolescents and their parents can be protective for suicidal behaviour among them [107]. Therefore, communication training as well psychoeducation programs for parents should be offered as preventive measures for families with adolescents, who have an increased risk of suicidal and self-harming behaviour, such as adolescents with migration background. A population-based intervention program for the Turkish community in Berlin reduced especially the suicide attempts of young Turkish women aged between 18 and 24 years living in Berlin, who showed the highest rate of suicide attempts at the beginning of the intervention programs. This population-based intervention programs offering inter alia professional culture and language sensitive telephone counselling in crisis, which has been made public with a citywide multilingual media campaign, substantially declined the rate of suicide attempts among young Turkish women [108]. Indeed culture and language-sensitive intervention programs, which actually reaches the target group sufficiently, are mostly quite effective at suicide prevention. Moreover, diversity-care interventions programs can increase the timely use of required professional support [109].

As shown in the case of Berlin mental health measures using diversity-care intervention programs can be relatively effective in preventing suicidal behaviour. Therefore, similarly to the above-mentioned mental health measures conducted in Berlin, also in Austria multilingual suicide prevention campaigns using population-based and community-based intervention programs, should be established in the health care system to prevent suicidal behaviour among youths with and without migration background.

Additionally further longitudinal studies in larger study samples focusing on the impact of acculturation stress on the suicidal behaviour of youths with a migration background are needed especially in countries with a high population of migrants.

## Conclusion

To treat migrant populations adequately and to decrease delays leading to often belated or even too late emergency care consultations instead of early interventions, culture and language-sensitive preventive patient-centred care is required [30, 102, 103, 110–113]. Low-barrier culture sensitive preventive measures for youths should be established in natural settings like in schools, to improve prevention of mental health problems including especially suicidal behaviour [111–114], considering also the differences in disclosure and help-seeking behaviours observed in our and in similar studies. Additionally multilingual and easily accessible “psycho-education programs” for migrants should be offered to reduce cultural barriers and to increase utilisation of mental health care offers. Such “psycho-education programs” may also be helpful to overcome the cultural conflicts within migrant families and between migrant generations [111–113]. Stigma of mental health problems and options for conflict resolution in intra-family conflicts might have to be addressed especially in traditional families by a comprehensive interdisciplinary approach in family care.

## Limitations

1. As the present study is based on a retrospective analysis; only the available information, which was recorded in the patient files could be used, though records in the participating centres were mostly based on similar standardised models and therefore usually quite complete. Still, some limitations apply.
2. Socio-economic status of individuals plays a crucial role in their suicidal behaviour [49, 115]. Unfortunately in our study this information was not recorded in most of the patient files, therefore the socio-economic status could have interacted with other migration-related effects and might have been missing in our statistical analysis. It might further differ between the participating sites.
3. Acculturative stress impairs the physical as well as the mental health of migrants [44, 45, 116]. In the present study, the acculturation stress of migrant youths may influence the transcultural differences concerning psychological disorders. Unfortunately, in our study the acculturation stress was not recorded because of the retrospective character and the limitation of standard records in our study.
4. As we compared suicide attempters with a Turkish migration background with their non-migrant peers living in Vienna, Berlin and Istanbul, who had an acute treatment in the respective hospitals, our results cannot be generalized to the whole population as well to other groups of migrants.

## Data Availability

All data and material are available at the Department of Child and Adolescent Psychiatry at the Medical University Vienna.

## References

[1] World Health Organization (2019) Suicide prevention. https://www.who.int/health-topics/suicide#tab=tab_1. Accessed 10 No 2019

[2] World Health Organization (2018). Global Health Estimates 2016: deaths by cause, age, sex, by country and by region, 2000–2016.

[3] Kapusta N, Grabenhofer-Eggerth A, Blüml V, Klein J, Baus N, Huemer J (2014) Suizid und Suizid-prävention in Österreich. Basisbericht 2013. Bundesministerium für Gesundheit, Wien

[4] Eskin M (2012). The role of childhood sexual abuse, childhood gender nonconformity, self-esteem and parental attachment in predicting suicide ideation and attempts in Turkish young adults. Suicidol Online.

[5] Eskin M, Voracek M, Stieger S, Altinyazar V (2011). A cross-cultural investigation of suicidal behavior and attitudes in Austrian and Turkish medical students. Soc Psychiatry Psychiatr Epidemiol.

[6] Skala K, Kapusta ND, Schlaff G, Unseld M, Erfurth A, Lesch OM, Walter H, Akiskal KK, Akiskal HS (2012). Suicidal ideation and temperament: an investigation among college students. J Affect Disord.

[7] Toprak S, Cetin I, Guven T, Can G, Demircan C (2011). Self-harm, suicidal ideation and suicide attempts among college students. Psychiatry Res.

[8] Zhang X, Wang H, Xia Y, Liu X, Jung E (2012). Stress, coping and suicide ideation in Chinese college students. J Adolesc.

[9] Mental Health Foundation (2018) Suicide. www.mentalhealth.org.uk/a-to-z/s/suicide. Accessed 15 Oct 2018

[10] World Health Organization (2016) Suicide rate estimates, age-standardized Estimates by country. http://apps.who.int/gho/data/view.main.MHSUICIDEASDRv?lang=en. Accessed 15 Apr 2019

[11] Statistik Austria (2018) Todesursachen im Überblick. http://www.statistik-austria.at/web_de/statistiken/menschen_und_gesellschaft/gesundheit/todesursachen/todesursachen_im_ueberblick/index.html. Accessed 15 May 2019

[12] Donath C, Graessel E, Baier D, Bleich S, Hillemacher T (2014). Is parenting style a predictor of suicide attempts in a representative sample of adolescents?. BMC Pediatr.

[13] Kaess M, Parzer P, Haffner J, Steen R, Roos J, Klett M, Brunner R, Resch F (2011). Explaining gender differences in non-fatal suicidal behaviour among adolescents: a population-based study. BMC Public Health.

[14] Plener PL, Libal G, Keller F, Fegert JM, Muehlenkamp JJ (2009). An international comparison of adolescent non-suicidal self-injury (NSSI) and suicide attempts: Germany and the USA. Psychol Med.

[15] Mars B, Heron J, Klonsky ED, Moran P, O'Connor RC, Tilling K, Wilkinson P, Gunnell D (2019). Predictors of future suicide attempt among adolescents with suicidal thoughts or non-suicidal self-harm: a population-based birth cohort study. Lancet Psychiatry.

[16] Townsend E (2019). Time to take self-harm in young people seriously. Lancet Psychiatry.

[17] Donald M, Dower J, Correa-Velez I, Jones M (2006). Risk and protective factors for medically serious suicide attempts: a comparison of hospital-based with population-based samples of young adults. Aust N Z J Psychiatry.

[18] Florenzano UR, Valdes CM, Caceres CE, Santander RS, Aspillaga HC, Musalem AC (2011). Relation between suicidal ideation and parenting styles among a group of Chilean adolescents. Rev Med Chil.

[19] World Health Organisation (2017) Suicide—Fact sheet. http://www.who.int/mediacentre/factsheets/fs398/en/. Accessed 10 Oct 2019

[20] Grube M (2004). Suizidversuche von Migranten in der Akutpsychiatrie. Nervenarzt.

[21] van Bergen DD, Smit JH, van Balkom AJ, van Ameijden E, Saharso S (2008). Suicidal ideation in ethnic minority and majority adolescents in Utrecht, the Netherlands. Crisis.

[22] Yilmaz TA, Riecher-Rössler A (2008). Suizidversuche in der ersten und zweiten Generation der Immigranten aus der Türkei. Neuropsychiatrie.

[23] Akkaya-Kalayci T, Popow C, Waldhor T, Ozlu-Erkilic Z (2015). Impact of religious feast days on youth suicide attempts in Istanbul, Turkey. Neuropsychiatr.

[24] Plener PL, Munz LM, Allroggen M, Kapusta ND, Fegert JM, Groschwitz RC (2015). Immigration as risk factor for non-suicidal self-injury and suicide attempts in adolescents in Germany. Child Adolesc Psychiatry Ment Health.

[25] Abebe DS, Lien L, Hjelde KH (2014). What we know and don't know about mental health problems among immigrants in Norway. J Immigr Minor Health.

[26] Giallo R, Riggs E, Lynch C, Vanpraag D, Yelland J, Szwarc J, Duell-Piening P, Tyrell L, Casey S, Brown SJ (2017). The physical and mental health problems of refugee and migrant fathers: findings from an Australian population-based study of children and their families. BMJ Open.

[27] Sturm G (2003). Die transkulturelle Psychotherapie nach Marie Rose Moro. Psychosozial.

[28] Michel PO, Lundin T, Larsson G (2007). Suicide rate among former Swedish peacekeeping personnel. Mil Med.

[29] Braun M, BRecchi E, Berger PAWA (2008). Keine Grenzen, mehr Opportunitäten?. Transnationalisierung sozialer Ungleichheit.

[30] Lindert J, Schouler-Ocak M, Heinz A, Priebe S (2008). Mental health, health care utilisation of migrants in Europe. Eur Psychiatry.

[31] Rezapour H, Zapp M (2011) Muslime in der Psychotherapie Vandenhoeck and Ruprecht GmbH & Co. KG, Göttingen

[32] Webb RT, Antonsen S, Mok PL, Agerbo E, Pedersen CB (2015). National cohort study of suicidality and violent criminality among danish immigrants. PLoS ONE.

[33] Bourque F, van der Ven E, Malla A (2011). A meta-analysis of the risk for psychotic disorders among first- and second-generation immigrants. Psychol Med.

[34] Ceri V, Ozlu-Erkilic Z, Ozer U, Kadak T, Winkler D, Dogangun B, Akkaya-Kalayci T (2017). Mental health problems of second generation children and adolescents with migration background. Int J Psychiatry Clin Pract.

[35] Leavey G, Hollins K, King M, Barnes J, Papadopoulos C, Grayson K (2004). Psychological disorder amongst refugee and migrant schoolchildren in London. Soc Psychiatry Psychiatr Epidemiol.

[36] Loue S, Sajatovic M (2012). Encyclopedia of immigrant health.

[37] Lu Y (2010). Rural-urban migration and health: evidence from longitudinal data in Indonesia. Soc Sci Med.

[38] Özlü-Erkilic, Klar S, Trinkl L (2015). Ent-Fremdungen—Transkulturelle Aspekte in der psychotherapeutischen Betreuung und Begleitung von türkischsprachigen Migrant_innen in Österreich. Diagnose: Besonderheit, Systemische Psychotherapie an den Rändern der Norm.

[39] Saraiva Leao T, Sundquist J, Johansson LM, Johansson SE, Sundquist K (2005). Incidence of mental disorders in second-generation immigrants in sweden: a four-year cohort study. Ethn Health.

[40] Skala K, Bruckner T (2014). Beating the odds: an approach to the topic of resilience in children and adolescents. Neuropsychiatr.

[41] Donath C, Bergmann MC, Kliem S, Hillemacher T, Baier D (2019). Epidemiology of suicidal ideation, suicide attempts, and direct self-injurious behavior in adolescents with a migration background: a representative study. BMC Pediatr.

[42] Carrasco-Sanz A, Leiva-Gea I, Martin-Alvarez L, Del Torso S, van Esso D, Hadjipanayis A, Kadir A, Ruiz-Canela J, Perez-Gonzalez O, Grossman Z (2018). Migrant children's health problems, care needs, and inequalities: European primary care paediatricians' perspective. Child Care Health Dev.

[43] Bhugra D (2004). Migration and mental health. Acta Psychiatr Scand.

[44] Gutmann MT, Aysel M, Ozlu-Erkilic Z, Popow C, Akkaya-Kalayci T (2019). Mental health problems of children and adolescents, with and without migration background, living in Vienna, Austria. Child Adolesc Psychiatry Ment Health.

[45] Özlü-Erkilic Z, Winkler D, Popow C, Zesch H, Akkaya-Kalayci T (2016). A comparative study of Turkish-speaking migrants and natives living in Vienna/Austria concerning their life satisfaction—with a particular focus on satisfaction regarding their health. Int J Migr Health Soc Care.

[46] Sluzki CE, Hegemann TSR (2006). Psychologische Phasen der Migration und ihre Auswirkungen. Transkulturelle Psychiatrie Konzepte für die Arbeit mit Menschen aus anderen Kulturen.

[47] Wang J, Liu K, Zheng J, Liu J, You L (2017). Prevalence of mental health problems and associated risk factors among rural-to-urban migrant children in Guangzhou.

[48] Montesinos AH, Bromand Z, Aichberger MC, Temur-Erman S, Yesil R, Rapp MA, Heinz A, Schouler-Ocak A (2010). Suizid und suizidales Verhalten bei Frauen mit türkischem Migrationshintergrund. Z Psychiatr Psychol Psychother.

[49] Ceccherini-Nelli A, Priebe S (2011). Economic factors and suicide rates: associations over time in four countries. Soc Psychiatry Psychiatr Epidemiol.

[50] Akkaya-Kalayci T, Popow C, Waldhor T, Winkler D, Ozlu-Erkilic Z (2017). Psychiatric emergencies of minors with and without migration background. Neuropsychiatrie.

[51] Austria S (2012). Statistisches Jahrbuch für migration and integration 2012.

[52] Razum O, Zeeb H (2004). Suizidsterblichkeit unter Türkinnen und Türken in Deutschland. Nervenarzt.

[53] Statistisches Bundesamt (2017) Bevölkerungsstand. https://www.destatis.de/DE/Themen/Gesellschaft-Umwelt/Bevoelkerung/Bevoelkerungsstand/Tabellen/zensus-geschlecht-staatsangehoerigkeit-2018.html. Accessed 10 May 2019

[54] Norredam M, Krasnik A, Petersen JH (1999). Access to Danish health care by immigrant women. Access to hospital care among immigrant women with breast cancer compared with Danish women. Ugeskr Laeger.

[55] Sanz B, Torres AM, Schumacher R (2000). Sociodemographic characteristics and use of health services by the immigrant population residing in a district of the Community of Madrid. Aten Primaria.

[56] European Commission (2018) Migration and Home Affairs. https://ec.europa.eu/home-affairs/what-we-do/networks/european_migration_network/glossary_search/person-migratory-background_en. Accessed 22 Jul 2019

[57] Statistik Austria (2013) Bevölkerung am 1.1.2013 nach zusammengefasstem Geburtsland, Geschlecht und Altersgruppen 2013. www.statistikaustria.at. Accessed 8 Apr 2019

[58] Nock MK, Borges G, Bromet EJ, Cha CB, Kessler RC, Lee S (2008). Suicide and suicidal behavior. Epidemiol Rev.

[59] Elisei S, Verdolini N, Anastasi S (2012). Suicidal attempts among Emergency Department patients: one-year of clinical experience. Psychiatr Danub.

[60] Monnin J, Thiemard E, Vandel P, Nicolier M, Tio G, Courtet P, Bellivier F, Sechter D, Haffen E (2012). Sociodemographic and psychopathological risk factors in repeated suicide attempts: gender differences in a prospective study. J Affect Disord.

[61] Naidoo SS, Schlebusch L (2014). Sociodemographic characteristics of persons committing suicide in Durban, South Africa: 2006–2007. Afr J Prim Health Care Fam Med.

[62] Rancic N, Ignjatovic Ristic D, Radovanovic S, Kocic S, Radevic S (2012). Sociodemographic and clinical characteristics of hospitalized patients after suicide attempt: a twenty-year retrospective study. Med Glas (Zenica).

[63] Dilling H, Mombour W, Schmidt MH (2004) International classification of mental disorders: ICD-10 Chapter V (F). Diagnostic guide-lines. Huber, Bern

[64] World Health Organisation (1993) The ICD-10 classification of mental and behavioural disorders. Clinical descriptions and diagnostic guidelines. WHO, Genf

[65] Akkaya-Kalayci T, Kapusta ND, Waldhor T, Bluml V, Poustka L, Ozlu-Erkilic Z (2017). The association of monthly, diurnal and circadian variations with suicide attempts by young people. Child Adolesc Psychiatry Ment Health.

[66] Akkaya-Kalayci T, Popow C, Winkler D, Bingol RH, Demir T, Ozlu Z (2015). The impact of migration and culture on suicide attempts of children and adolescents living in Istanbul. Int J Psychiatry Clin Pract.

[67] Akkaya-Kalayci T, Vyssoki B, Winkler D, Willeit M, Kapusta ND, Dorffner G, Ozlu-Erkilic Z (2017). The effect of seasonal changes and climatic factors on suicide attempts of young people. BMC Psychiatry.

[68] Ozdel O, Varma G, Atesci FC, Oguzhanoglu NK, Karadag F, Amuk T (2009). Characteristics of suicidal behavior in a Turkish sample. Crisis.

[69] Ozlu-Erkilic Z, Wenzel T, Kothgassner OD, Akkaya-Kalayci T (2020). Transcultural differences in risk factors and in triggering reasons of suicidal and self-harming behaviour in young people with and without a migration background. Int J Environ Res Public Health.

[70] Athani P, Hugar BS, Harish S, Girishchandra YP (2017). Pattern of unnatural deaths among children: an autopsy study. Med Leg J.

[71] Organisation WH (2019). Suicide in the world: global health estimates.

[72] World Health Organisation (1999). Figures and facts about suicide.

[73] Akkaya-Kalayci T, Kapusta ND, Winkler D, Kothgassner OD, Popow C, Ozlu-Erkilic Z (2018). Triggers for attempted suicide in Istanbul youth, with special reference to their socio-demographic background. Int J Psychiatry Clin Pract.

[74] Evans E, Hawton K, Rodham K (2004). Factors associated with suicidal phenomena in adolescents: a systematic review of population-based studies. Clin Psychol Rev.

[75] O'Donnell L, O'Donnell C, Wardlaw DM, Stueve A (2004). Risk and resiliency factors influencing suicidality among urban African American and Latino youth. Am J Community Psychol.

[76] Kwok SY, Shek DT (2010). Hopelessness, parent-adolescent communication, and suicidal ideation among Chinese adolescents in Hong Kong. Suicide Life Threat Behav.

[77] Martin G, Rozanes P, Pearce C, Allison S (1995). Adolescent suicide, depression and family dysfunction. Acta Psychiatr Scand.

[78] Samm A, Tooding LM, Sisask M, Kolves K, Aasvee K, Varnik A (2010). Suicidal thoughts and depressive feelings amongst Estonian schoolchildren: effect of family relationship and family structure. Eur Child Adolesc Psychiatry.

[79] Zaborskis A, Sirvyte D, Zemaitiene N (2016). Prevalence and familial predictors of suicidal behaviour among adolescents in Lithuania: a cross-sectional survey 2014. BMC Public Health.

[80] Shrestha AK, Ozlu-Erkilic Z, Popow C, Ohmann S, Akkaya-Kalayci T (2019). Transcultural differences of psychologically traumatised children and adolescents. Neuropsychiatr.

[81] Tompkins D, Galbraith D, Tompkins P (2010). Universalism, particularism and cultural self-awareness: a comparison of American and Turkish university students. J Int Bus Cult Stud.

[82] Hofstede G (1980). Culture’s consequences: international differences in work-related values.

[83] Tyyskä V (2008). Parents and teens in immigrant families: cultural influences and material pressures. Can Divers.

[84] Ferber L, Köster I, Celayir-Erdogan N (2003). Türkische und deutsche Hausarztpatienten-Erkrankungen, Arzneimittelerwartungen und Verordnungen. Das Gesundheitswesen.

[85] von Ferber L, Koster I, Celayir-Erdogan N (2003). Turkish and German patients of general practitioners–diseases, drug expectations and drug prescriptions. Gesundheitswesen.

[86] Garlow SJ, Rosenberg J, Moore JD, Haas AP, Koestner B, Hendin H, Nemeroff CB (2008). Depression, desperation, and suicidal ideation in college students: results from the American Foundation for Suicide Prevention College Screening Project at Emory University. Depress Anxiety.

[87] Hawton K, Casanas ICC, Haw C, Saunders K (2013). Risk factors for suicide in individuals with depression: a systematic review. J Affect Disord.

[88] McHugh CM, Chun Lee RS, Hermens DF, Corderoy A, Large M, Hickie IB (2019). Impulsivity in the self-harm and suicidal behavior of young people: a systematic review and meta-analysis. J Psychiatr Res.

[89] World Health Organisation (2010) Fact sheets. https://www.who.int/news-room/fact-sheets/detail/suicide. Accessed 31 Oct 2011

[90] Hofbauer S, Schütz B, Biffl G, John M, Perchinig B, Bock-Shappelwein J, Ferreri C, Carl Y, Moongananiyil S, Diaz N (2004) Der Einfluss von Immigration auf die Österreichische Gesellschaft. Österreichischer Beitrag im Rahmen der europaweiten Pilotstudie “The Impact of Immigration on Europes Societies. IOM International Organisation for Migration, Wien

[91] Hunn K (2002). Asymmetrische Beziehungen: Türkische , Gastarbeiter‘ zwischen Heimat und Fremde. Vom deutsch-türkischen Anwerbeabkommen bis zum Anwerbestopp (1961–1973). Archiv für Sozialgeschichte.

[92] Statistic Austria (2018) Bevölkerung in Privathaushalten nach Migrationshintergrund. https://www.statistik.at/web_de/statistiken/menschen_und_gesellschaft/bevoelkerung/bevoelkerungsstruktur/bevoelkerung_nach_migrationshintergrund/index.html Accessed 5 Apr

[93] Ceballos M, Palloni A (2010). Maternal and infant health of Mexican immigrants in the USA: the effects of acculturation, duration, and selective return migration. Ethn Health.

[94] Eskin M, Poyrazli S, Janghorbani M, Bakhshi S, Carta MG, Moro MF, Tran US, Voracek M, Mechri A, Aidoudi K, Hamdan M, Nawafleh H, Sun JM, Flood C, Phillips L, Yoshimasu K, Tsuno K, Kujan O, Harlak H, Khader Y, Shaheen A, Taifour S (2019). The role of religion in suicidal behavior, attitudes and psychological distress among university students: a multinational study. Transcult Psychiatry.

[95] Harry Hui C, Triandis HC (1986). Individualism-collectivism: a study of cross-cultural researchers. J Cross Cult Psychol.

[96] Turkish Statistical Institute (2018) Nüfus istatistigi. http://www.tuik.gov.tr/UstMenu.do?metod=temelist. Accessed 15 Jun 2019

[97] Eylem O, van Straten A, Bhui K, Kerkhof AJ (2015). Protocol: reducing suicidal ideation among Turkish migrants in the Netherlands and in the UK: effectiveness of an online intervention. Int Rev Psychiatry.

[98] Galletly C, Burton C (2011). Improving medical student attitudes towards people with schizophrenia. Aust N Z J Psychiatry.

[99] Papadopoulos C, Foster J, Caldwell K (2013). ‘Individualism-collectivism’ as an explanatory device for mental illness stigma. Community Ment Health J.

[100] Pettigrew TF, Tropp LR (2006). A meta-analytic test of intergroup contact theory. J Pers Soc Psychol.

[101] Renzaho A, McCabe M, Sainsbury W (2011). Parenting, role reversals and the preservation of cultural values among Arabic speaking migrant families in Melbourne, Australia. Int J Intercult Relat.

[102] Akkaya-Kalayci T (2017). Die Bedeutung transkultureller Kompetenz in der Psychiatrie. Kasuistik eines 17-jährigen Flüchtlings aus Syrien. Psychopraxis neuropraxis.

[103] Binder-Fritz C, Akkaya-Kalayci T (2014). Soziokulturelle und psychische Aspekte der Migration: Vorstellung des Universitätslehrgangs „Transkulturelle Medizin & Diversity Care. Wien Klin Wochenschr.

[104] Anson O (2011). Inequality in the access to preventive health care: the case of immigrants in Belgium. Arch Public Health.

[105] Levecque K, Lodewyckx I, Bracke P (2009). Psychological distress, depression and generalised anxiety in Turkish and Moroccan immigrants in Belgium: a general population study. Soc Psychiatry Psychiatr Epidemiol.

[106] Brent DA, McMakin DL, Kennard BD, Goldstein TR, Mayes TL, Douaihy AB (2013). Protecting adolescents from self-harm: a critical review of intervention studies. J Am Acad Child Adolesc Psychiatry.

[107] Radde S, Gutwinski S, Stuke F, Fuchs A, Schouler-Ocak M, Bermpohl F, Henssler J (2018). Suicidal tendencies in adolescence: dysfunctional familiar communication as risk factor. Nervenarzt.

[108] Aichberger MC, Heredia Montesinos A, Bromand Z, Yesil R, Temur-Erman S, Rapp MA, Heinz A, Schouler-Ocak M (2015). Suicide attempt rates and intervention effects in women of Turkish origin in Berlin. Eur Psychiatry.

[109] Schouler-Ocak M, Montesinos AH, Aichberger MC (2014). Suizidprävention bei Frauen mit türkischem Migrationshintergrund. Ärztliche Psychother.

[110] Jaeger FN, Hossain M, Kiss L, Zimmerman C (2012). The health of migrant children in Switzerland. Int J Public Health.

[111] Akkaya-Kalayc T, Dervic K, Friedrich MH (2007). Viennese transcultural outpatient clinic for child psychiatry. Eur Psychiatry.

[112] Akkaya-Kalayci T (2011) The impact of special psychiatric services on migrant children and their families. In: World sICoCPitFs (ed) Migration challenges and mental health. France

[113] Akkaya-Kalayci T (2011) The special needs of migrant children and their families in mental health care services. In: The 3rd world congress of Cultural Psychiatry. London, England

[114] Christodulu KV, Lichenstein R, Weist MD, Shafer ME, Simone M (2002). Psychiatric emergencies in children. Pediatr Emerg Care.

[115] Zivin K, Paczkowski M, Galea S (2011). Economic downturns and population mental health: research findings, gaps, challenges and priorities. Psychol Med.

[116] John B, Segall M, Kagitcibasi C (1997) Handbook of cross-cultural psychology. Social behaviour and applications

